# Dermal Injection of Recombinant Filaggrin-2 Ameliorates UVB-Induced Epidermal Barrier Dysfunction and Photoaging

**DOI:** 10.3390/antiox13081002

**Published:** 2024-08-19

**Authors:** Lu Li, Yuan Liu, Ruxue Chang, Tao Ye, Ziyi Li, Rufei Huang, Zhaoyang Wang, Jingxian Deng, Huan Xia, Yan Yang, Yadong Huang

**Affiliations:** 1 Department of Cell Biology, Jinan University, Guangzhou 510632, China; lilu2022@stu2022.jnu.edu.cn (L.L.); yuanliu@stu2021.jnu.edu.cn (Y.L.); chang2023@stu.jnu.edu.cn (R.C.); taoyhust@stu2022.jnu.edu.cn (T.Y.); sophie12@stu2022.jnu.edu.cn (R.H.); wzy1003@stu2021.jnu.edu.cn (Z.W.); dengjingxian@stu2021.jnu.edu.cn (J.D.); 2 State Key Laboratory of Bioactive Molecules and Druggability Assessment, Jinan University, Guangzhou 510632, China; 3 TYRAN Cosmetics Innovation Research Institute, Jinan University, Guangzhou 511447, China; lzy2023@stu2023.jnu.edu.cn (Z.L.); xiahuan@stu2019.jnu.edu.cn (H.X.); 4 National Engineering Research Center of Genetic Medicine, Guangzhou 510632, China; 5 Guangdong Province Key Laboratory of Bioengineering Medicine, Guangzhou 510632, China

**Keywords:** epidermal barrier, FLG2, UV radiation, keratinocyte differentiation, photoaging

## Abstract

The epidermal barrier is vital for protecting the skin from environmental stressors and ultraviolet (UV) radiation. Filaggrin-2 (FLG2), a critical protein in the stratum corneum, plays a significant role in maintaining skin barrier homeostasis. However, the precise role of FLG2 in mitigating the adverse effects of UV-induced barrier disruption and photoaging remains poorly understood. In this study, we revealed that UVB exposure resulted in a decreased expression of FLG2 in HaCaT keratinocytes, which correlated with a compromised barrier function. The administration of recombinant filaggrin-2 (rFLG2) enhanced keratinocyte differentiation, bolstered barrier integrity, and offered protection against apoptosis and oxidative stress induced by UVB irradiation. Furthermore, in a UV-induced photodamage murine model, the dermal injection of rFLG2 facilitated the enhanced restoration of the epidermal barrier, decreased oxidative stress and inflammation, and mitigated the collagen degradation that is typical of photoaging. Collectively, our findings suggested that targeting FLG2 could be a strategic approach to prevent and treat skin barrier dysfunction and combat the aging effects associated with photoaging. rFLG2 emerges as a potentially viable therapy for maintaining skin health and preventing skin aging processes amplified by photodamage.

## 1. Introduction

The skin acts as a fundamental defense against environmental dangers and is essential for minimizing water loss and fending off pathogens [1]. The intact epidermal barrier, central to this defense, dynamically protects against transepidermal water loss and microbial invasion [1]. Compromised by genetics, environmental conditions, harsh substances, or ultraviolet (UVA and UVB) radiation, the barrier is susceptible to disorders such as atopic dermatitis (AD) [2], ichthyosis [3], and photoaging [4], making treatment and management a complex endeavor.

The structural integrity of the epidermal barrier is reliant upon the correct differentiation of keratinocytes, culminating in the formation of a fully matured stratum corneum (SC), the outermost layer of the epidermis [5,6]. This differentiation process begins in the stratum basale (SB), and as keratinocytes progress toward the stratum corneum, they undergo a series of well-defined morphological changes and produce specific molecular markers indicative of their differentiation status [7,8]. In the stratum basale layer, keratinocytes predominantly express keratins KRT5 and KRT14 [9]. Upon entering the stratum spinosum (SS), early differentiation markers such as KRT1 and KRT10 are produced [10], together with the essential constituents of the cornified envelope, such as involucrin (IVL) and transglutaminase-1 (TGM1) [6,11]. Advancing to the stratum granulosum (SG), keratinocytes express late differentiation markers, including the envelope proteins loricrin (LOR) and profilaggrin (proFLG) [8]. At this stage, TGM1 catalyzes the cross-linking of involucrin, loricrin, and other structural proteins, giving rise to the cornified envelope, vital for the epidermal barrier function [12]. Tight junctions (TJs) within the SG, critical to barrier efficacy, consist of transmembrane proteins such as claudins, occludin, and junctional adhesion molecules that anchor to cytoplasmic proteins, exemplified by zonula occludens-1 (ZO-1) [13]. Pathological examinations, particularly in atopic dermatitis, reveal a reduced expression of claudin-1 (CLDN1), leading to a compromised barrier function [14,15]. In vitro models suggested that the upregulation of tight junction components, such as claudins, ZO-1, and occludin, could potentially restore and enhance barrier function [16,17], suggesting a promising direction for therapeutic strategies.

Filaggrin-2 (FLG2) is a member of the S100 fused-type protein family, sharing structural and expression similarities with proFLG, a crucial player in the keratinization process [18]. It is prominently expressed in the basal layer of the epidermis, with particularly high levels in the granular layer and the stratum corneum [19]. Within the stratum corneum, filaggrin (FLG) undergoes deimination by peptidyl arginine deiminases, resulting in a neutral charge that allows it to detach from keratins and to be broken down into free amino acids through the action of various proteases, including caspase-14, bleomycin hydrolase, and calpain-1 [20]. These amino acids, such as pyrrolidone carboxylic acid and urocanic acid, form the natural moisturizing factor (NMF) and play a critical role in skin hydration, pH balance, photoprotection, and immune modulation [21,22]. However, the close relationship between FLG and FLG2 suggests that these two proteins may play similar roles in skin barrier function [18,23]. A model of acute barrier disruption induced by acetone treatment in murine skin showed reduced FLG2 expression at the protein level, as confirmed by immunohistochemical analyses [19]. In vitro studies utilizing reconstructed human epidermal models revealed that the knockdown of FLG2 with shRNA led to decreased levels of NMF components, including urocanic acid and pyrrolidone carboxylic acid, affecting their proteolytic maturation [23]. These amino acids are vital for UV absorption, providing protection against UVB irradiation [24]. The down-regulation of FLG2 also resulted in a compacted stratum corneum with parakeratosis, abnormal intracellular vesicles, and an elevated surface pH, indicating a compromised barrier function [23,25]. Additionally, the C-terminal domain of the FLG2 protein has shown antibacterial activity against Gram-negative bacteria, inhibiting their replication, and suggesting a potential role as an antimicrobial agent targeting the Pseudomonas species [26]. These findings underscore the importance of FLG2 in maintaining the integrity of the epidermal barrier and in antimicrobial defense, while its potential anti-photoaging effects remain to be fully explored.

Our study revealed the effects of UVB radiation on HaCaT cells, demonstrating that UVB-triggered cellular senescence, reduced FLG2 expression and hampered skin barrier function. Furthermore, using recombinant filaggrin-2 (rFLG2) to treat UVB-induced skin damage validated its role in preserving the skin barrier integrity and alleviating photoaging. These findings suggested that targeting FLG2 could play a significant role in preventing the onset and progression of skin barrier deterioration and photoaging.

## 2. Materials and Methods

### 2.1. The Animals

Six-week-old female Kunming mice were purchased from the experimental Animal Center of Guangdong Province, China. Before the start of the experiments, mice were permitted to acclimate for one week in a temperature and humidity-controlled facility (22 ± 1 °C and 55 ± 5% relative humidity) with a light/dark cycle of 12 h and provided access to food and tap water. All animals were housed in the specific -pathogen-free environment of the laboratory experiment research center, and all animal experiments were conducted in accordance with the Chinese Institutional Guidelines for Animal Care and Use and approved by the Laboratory Animal Welfare and Ethics Committee of Jinan University.

### 2.2. rFLG2 Expression and Purification

rFLG2 was procured from TYRAN BIOTECHNOLOGY CO. (Guangzhou, China). Briefly, the synthesized FLG2 gene fragment was cloned into the pET-3C vector, transformed into Escherichia coli BL21 (DE3), and sequenced (Sangon, Shanghai, China). After sequencing, the engineering strain pET3C-rFLG2/BL21 was induced to express the protein using IPTG. The bacteria were harvested, and the cell pellet was collected. rFLG2 was found to be present within the bacterial cells. These cells were sonicated, and the resulting lysate was centrifuged to separate the supernatant. The supernatant was then filtered through a membrane filter and subjected to purification using a nickel affinity column. Unbound impurities were removed by washing with a binding buffer (0.5 M NaCl, 50 mM phosphate buffer, 20 mM imidazole, 8 M urea), followed by a washing buffer (0.5 M NaCl, 50 mM PB, 80 mM imidazole). The bound protein was subsequently eluted using an elution buffer (0.5 M NaCl, 50 mM PB, 300 mM imidazole). To obtain high-purity rFLG2, the eluted protein was passed through a G25 desalting column to remove urea and imidazole. The final purified rFLG2 was collected for further use.

### 2.3. UVB Irradiation and Treatments

After a one-week acclimatization period, the mice were randomly assigned to six groups, with eight mice in each group: a control group (no UVB-irradiation), a UVB-irradiated group, a UVB-irradiated group treated with PBS, a UVB-irradiated group treated with different concentrations of rFLG2 (0.25 mg/mL, 0.5 mg/mL, 1.0 mg/mL). The hair on the back of the mice was removed using hair removal cream (ICE King). The following day, the mice were subjected to daily UVB exposure (16.6 mW/cm^2^) for 2 h every day, resulting in a daily UVB irradiation dose of 120 mJ/cm^2^. The irradiation dose was calculated using the following formula: dose (mJ/cm^2^) = exposure time (s) × intensity (mW/cm^2^). The entire irradiation period spanned 36 days, with supplemental hair removal performed before each irradiation session. For the treatment groups, the exposed dorsal skin of the animals received a weekly dermal injection of 300 μL rFLG2, evenly dotted using a microinjection apparatus.

### 2.4. Trans-Epithelial Electrical Resistance (TEER)

After establishing the HaCaT cells monolayer model, the electrodes were soaked in PBS and wiped with 75% alcohol. Subsequently, the electrodes were connected to an electrical resistance meter to measure the TEER in HaCaT cell monolayers. The TEER value of the cells was determined by calculating the difference between the resistance value measured in the chamber inoculated with cells and the chamber uninoculated with cells, referred to as the blank value.

### 2.5. Histopathology

After the experiment, the mice were sacrificed, and the enlarged skin covering the injection area and its subcutaneous tissue were quickly removed, rinsed with PBS, and fixed in 4% paraformaldehyde for 24 h. The tissue was then embedded in paraffin and cut into 5 μm. After deparaffinization and hydration, H&E staining was used to observe the distribution and the status of cells and matrix. Sirius red staining was used to distinguish different types of collagen in the skin matrix. Masson’s trichrome staining was used to observe the morphology and structure of collagen fibers. For any quantitative analysis based on histological staining, at least 5 different visual fields were measured.

### 2.6. Hydroxyproline Content Determination

Equal quantities of skin tissue were processed using a commercial hydroxyproline assay kit (Jiancheng Bio, Nanjing, China) according to the manufacturer’s instructions. The hydroxyproline content in each sample was determined by measuring the absorbance at 550 nm and expressed in micrograms of hydroxyproline per milligram of tissue.

### 2.7. Antioxidant Enzyme Activities

The detection of superoxide dismutase (SOD) activity and malondialdehyde (MDA) content was performed by spectrophotometry, using a commercially available assay kits (Solarbio, Beijing, China). Skin tissues were processed according to the manufacturer’s instructions, and the absorbance at 560 nm of the samples was measured using a spectrophotometer to calculate the SOD activity and MDA content.

### 2.8. Immunohistochemistry

The paraffin-embedded skin tissue was cut into 4 μm thick sections. After deparaffinization and hydration, the sections were incubated in a 3% endogenous peroxidase blocking solution for 10 min. Subsequently, the sections were blocked with 5% BSA for 1 h at room temperature, followed by overnight incubation with the primary antibody at 4 °C. On the subsequent day, the sections were incubated with secondary antibodies at 37 °C for 1 h, and DAB solution (Boster Biological Technology, Pleasanton, CA, USA) was added to enable visualization. Finally, after counterstaining with hematoxylin, the immunostained sections were observed under a microscope.

### 2.9. Cell Culture and Treatments

HaCaT cells were obtained from our laboratory and were maintained in DMEM supplemented with 10% fetal bovine serum, 100 units/mL penicillin, and 0.1 mg/mL streptomycin, in an incubator with a 37 °C humidified atmosphere containing 5% CO_2_. HaCaT cells were irradiated in the well plates. For UVB irradiation, the medium of HaCaT cells was replaced with PBS. The cells were exposed to 2 mJ/cm^2^ UVB for 2 min 30 s in the uncovered well plates. Subsequently, we replaced the PBS with DMEM containing rFLG2 (12.5, 25, 50 μg/mL), and continued culturing in the incubator for 24 h.

### 2.10. Cytotoxicity Assay

HaCaT cells were seeded in a 96-well plate at a density of 10,000 cells per well. Then, these cells were exposed to UVB according to a previously described protocol. Next day, CCK8 (10 μL/well) was applied for 1 h and measured at 490 nm.

### 2.11. ROS Determination

To detect the level of intracellular ROS generation, 5 × 10^5^ HaCaT cells were seeded in each well of the 6-well plate. After treatment, the cells were incubated with serum-free DMEM containing 10 μM DCFH-DA (Beyotime, Shanghai, China) at 37 °C for 20 min. Subsequently, the cells were digested and the fluorescence intensity was measured by flow cytometry (BD Biosciences, San Jose, CA, USA).

### 2.12. RNA Isolation and Real-Time Quantitative Polymerase Chain Reaction (RT-qPCR)

The total RNA of sampled tissue was extracted and isolated using TRIZOL reagent (Invitrogen, Carlsbad, CA, USA) according to the manufacturer’s instructions. An amount of 1 μg RNA was reverse transcribed into cDNA using the PrimeScript™ RT Master Mix (Takara, Otsu, Japan). RT-qPCR was performed on CFX-96 Real-Time System thermal cycler (Bio-Rad, Hercules, CA, USA) using the ChamQ SYBR qPCR Master Mix (Vazyme, Nanjing, China). Amplification efficiency was determined for each pair of primers using the cDNA template gradient dilution method to ensure approximately the same efficiency for amplification, allowing for accurate analysis using the 2^−ΔΔCt^ method. Table 1 lists the genes and primers used for the RT-qPCR analyses in this study.

### 2.13. Western Blot Assay

The treated cells were washed with PBS and lysed with RIPA buffer. The lysate was centrifuged at 12,000× *g* rpm for 30 min, and the supernatant was collected and quantified using a BCA protein assay kit (Thermo Fisher Scientific, Waltham, MA, USA). The isolated proteins were mixed with SDS loading buffer and boiled for 10 min. The protein samples were separated by SDS-PAGE and transferred to the PVDF membrane. The membrane was blocked with 5% skim milk at room temperature for 1 h, then incubated with the primary antibodies against ZO-1 (Abcam, Cambridge, UK), loricrin (Abcam, Cambridge, UK), caspase14 (Abcam, Cambridge, UK), involucrin (Invitrogen, Carlsbad, CA, USA), at 4 °C overnight. Subsequently, it was incubated with HRP-conjugated secondary antibodies (FudeBio, Hangzhou, China) at room temperature for 1 h. Finally, the immunoreactive bands were detected using the enhanced chemiluminescence (ECL) reagent.

### 2.14. Statistical Analysis

Data analysis was conducted using GraphPad Prism 8.0 software (San Diego, CA, USA). All data were expressed as means ± standard deviation. Student’s *t*-tests were used for comparisons between two samples, while comparisons among multiple groups were performed using one-way analysis of variance (ANOVA).

## 3. Results

### 3.1. UVB Can Induce Photoaging and FLG2 Reduction

**Figure 1:**
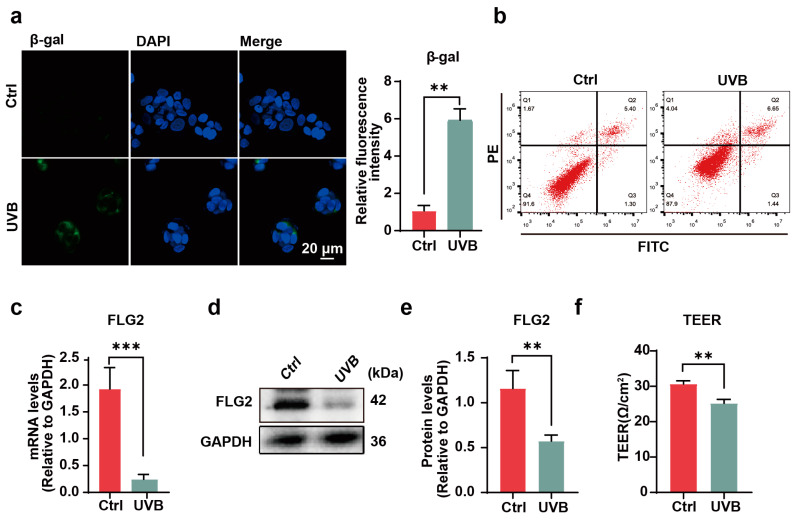
UVB can induce photoaging in HaCaTs. (**a**) UVB-treated cells were measured for senescence-associated β-galactosidase activity and the percentage of cells positive for β-galactosidase staining. Scale bar represents 20 μm. (**b**) Determining early apoptosis after UVB exposure by flow cytometry Annexin V/PI assay. (**c**) Filaggrin-2 (FLG2) gene expression changes in HaCaT cells before and after UVB irradiation. (**d**,**e**) Changes in FLG2 protein expression in HaCaT cells before and after UVB irradiation; GAPDH was used as the internal reference. (**f**) Transmembrane resistance was measured in monolayer cells after UVB irradiation. The data are representative of at least three independent experiments. Values are shown as mean ± SD. ** *p* < 0.01, *** *p* < 0.001.

To elucidate the effects of UVB radiation on HaCaT cell senescence and FLG2 expression, HaCaT cells were exposed to 2 mJ/cm^2^ UVB radiation. Cellular senescence was assessed by β-galactosidase staining. The UVB-irradiated cells exhibited a significant increase in β-galactosidase activity compared to the untreated control group (Figure 1a). Furthermore, flow cytometric analysis using Annexin V/PI staining revealed no significant induction of apoptosis (Figure 1b).

The subsequent evaluation of FLG2 expression demonstrated a noticeable downregulation at both transcriptional and translational levels in response to UVB exposure, as compared to the non-irradiated controls (Figure 1c–e). Moreover, following UVB irradiation, there was a notable decrease in the TEER of HaCaT cell monolayers, indicating a compromised integrity of the HaCaT cell barrier (Figure 1f).

Overall, these results suggested that UVB radiation induces senescence, suppresses FLG2 expression, and impairs the barrier integrity of HaCaT cells. The findings implied a possible association among FLG2, cellular integrity, and the senescence process in keratinocytes.

### 3.2. rFLG2 Promoted Keratinocyte Differentiation In Vitro

**Figure 2:**
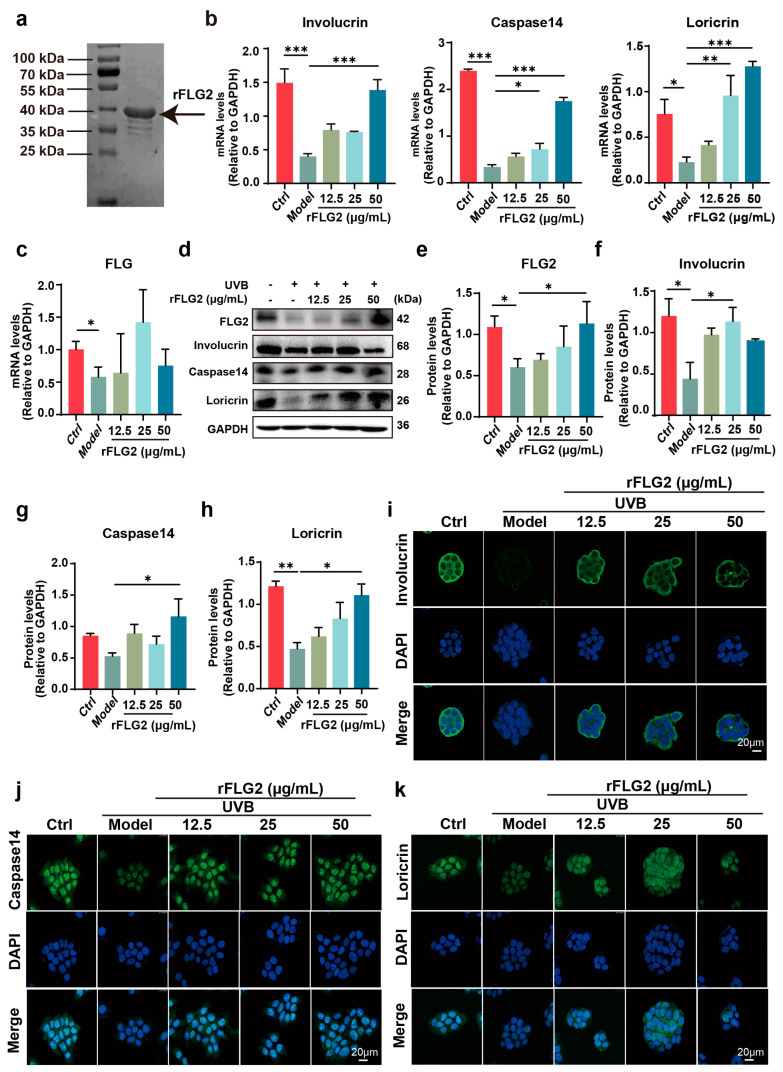
Recombinant filaggrin-2 (rFLG2) promoted keratinocyte differentiation in vitro. (**a**) The purified protein was separated by SDS-PAGE. The arrow shows the target protein (rFLG2). (**b**) mRNA expression of involucrin, loricrin, and caspase14 in HaCaTs was measured by RT-qPCR and normalized to GAPDH levels. (**c**) mRNA expression of filaggrin (FLG) in HaCaTs was measured by RT-qPCR and normalized to GAPDH levels. (**d**–**h**) HaCaTs were treated with rFLG2 in combination with UVB, and the protein level of the FLG2, involucrin, loricrin, and caspase14 were detected by Western blot; GAPDH was used as the internal reference. (**i**–**k**) The protein levels of involucrin, loricrin, and caspase14 in HaCaTs treated with UVB and rFLG2 were detected by immunofluorescence. Scale bar represents 20 μm. The data are representative of at least three independent experiments. Values are shown as mean ± SD. * *p* < 0.05, ** *p* < 0.01, *** *p* < 0.001.

Based on the above results, we used rFLG2 to examine whether FLG2 has the ability to rescue the impaired barrier function caused by UVB. The molecular weight of rFLG2 was determined by SDS-PAGE, and the result indicated that its molecular weight is approximately 40 kDa (Figure 2a). Then, we investigated the impact of rFLG2 on the differentiation of HaCaT cells induced by UVB radiation by assessing the expression levels of differentiation-related markers, including FLG, involucrin, loricrin, and caspase 14. The HaCaT cells exposed to UVB radiation were treated with varying concentrations of rFLG2 (12.5, 25, and 50 μg/mL). The results demonstrated that UVB exposure significantly reduced the mRNA expression of involucrin, caspase 14, and loricrin compared to the control group. In contrast, treatment with rFLG2 led to a dose-dependent increase in the expression of these genes, showing the most significant effect at a concentration of 50 μg/mL (Figure 2b). Furthermore, exposure to UVB significantly reduced the FLG mRNA levels, while treatment with different doses of rFLG2 slightly, although not significantly, slightly upregulated its expression (Figure 2c).

Next, the protein levels of FLG2, involucrin, loricrin, and caspase 14 were evaluated using Western blot analyses (Figure 2d). Consistent with the mRNA results, UVB irradiation caused a significant decrease in the protein expression of these markers compared to the control group. However, treatment with rFLG2 at concentrations of 25 μg/mL and 50 μg/mL significantly enhanced their protein levels (Figure 2e–h). Immunofluorescence staining further confirmed the elevated protein expressions of these proteins, indicating that rFLG2 effectively induced keratinocyte differentiation in vitro (Figure 2i–k).

### 3.3. rFLG2 Improved the Epidermal Barrier Formation in Keratinocytes

**Figure 3:**
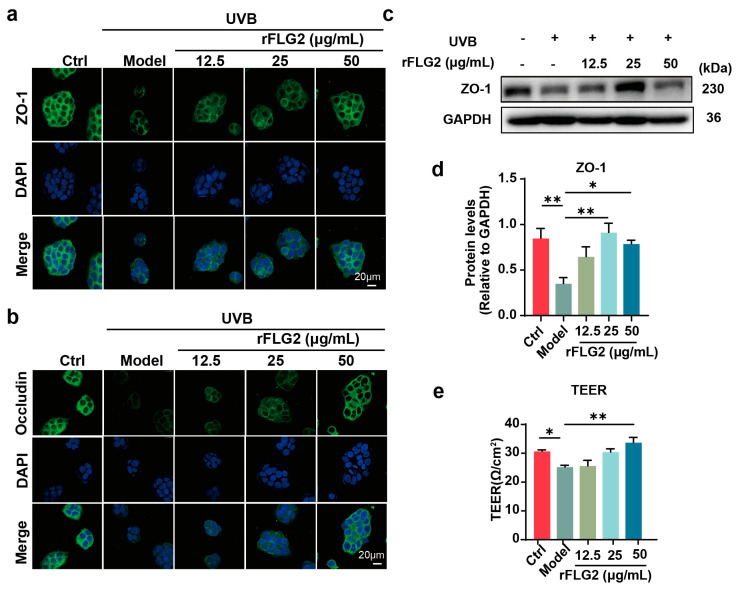
rFLG2 can alleviate UVB-induced damage to HaCaT barrier function. (**a**,**b**) Expression and distribution of zonula occludens-1 (ZO-1) and occludin proteins in HaCaTs were detected by immunofluorescence after being treated with rFLG2. Scale bar represents 20 μm. (**c**,**d**) HaCaTs were treated with rFLG2 in combination with UVB, and the expression level of ZO-1 was detected by Western blot; GAPDH was used as the internal reference. (**e**) Transmembrane resistance was measured in monolayer cells. The data are representative of at least three independent experiments. Values are shown as mean ± SD. * *p* < 0.05, ** *p* < 0.01.

Keratinocytes are the main cells that make up the epidermal structure and its barrier. We investigated the impact of rFLG2 treatment on the expression levels of TJ barrier-related proteins, including ZO-1 and occludin in the UVB-irradiated HaCaT cells. The immunofluorescence results showed that the expression of ZO-1 and occludin was significantly increased after treatment with rFLG2 at concentrations of 25 μg/mL and 50 μg/mL (Figure 3a,b). At the same time, the Western blot results showed that rFLG2 at the concentration of 25 μg/mL could significantly increase the protein level of ZO-1 (Figure 3c,d), which indicated that rFLG2 could counteract the UVB-induced reduction of TJ barrier-related proteins in keratinocytes. Additionally, we assessed the TEER value to analyze the effect of rFLG2 on the TJ barrier. UVB irradiation led to a significant reduction in the TEER value in HaCaT cells. Remarkably, the TEER values were significantly elevated at 48 h in the cells treated with 50 μg/mL rFLG2, indicating a strengthening of the TJ barrier and the recovery of impaired barrier function post-UVB exposure (Figure 3e).

### 3.4. rFLG2 Inhibited UVB-Induced Apoptosis Oxidative Stress in HaCaTs

**Figure 4:**
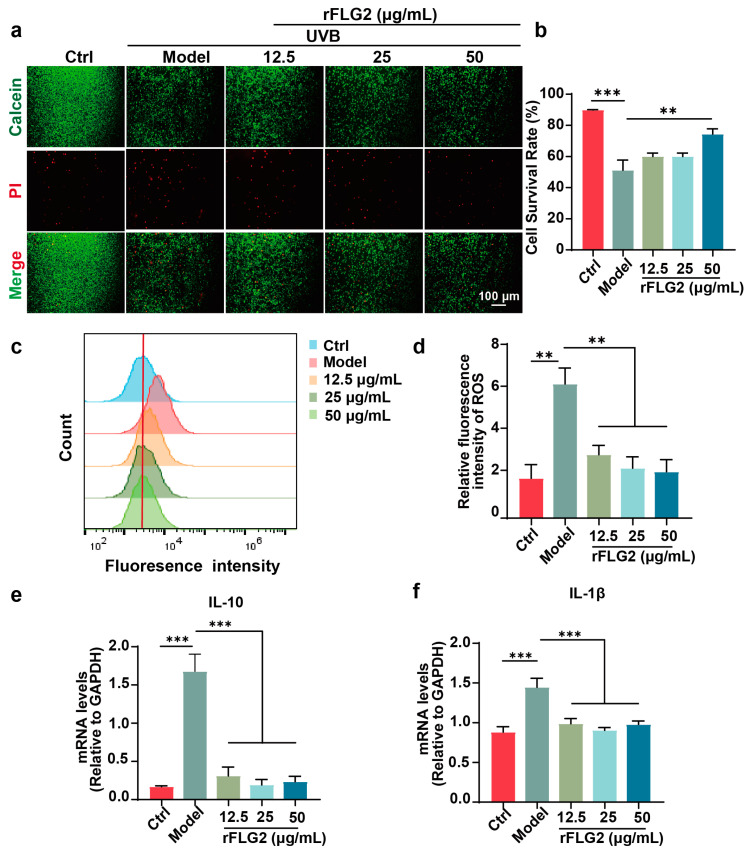
rFLG2 reduces UVB-induced oxidative stress. (**a**) Live cells were stained green, while dead cells were stained red with Calcein AM/PI. Scale bar represents 100 μm. (**b**) Fluorescence quantitative live and dead staining statistics. (**c**) Flow cytometry and analysis of ROS production in HaCaT cells after UVB and rFLG2 treatment. (**d**) Quantification of intracellular ROS levels. (**e**,**f**) Gene expression levels of inflammatory factors of IL-1β and IL-10 in HaCaT cells were measured by RT-qPCR and normalized to GAPDH levels. The data are representative of at least three independent experiments. Values are shown as mean ± SD. ** *p* < 0.01, *** *p* < 0.001.

To examine the influence of rFLG2 on UVB-induced senescence, HaCaT cells were exposed to UVB irradiation and then were treated with rFLG2 at concentrations of 12.5, 25, and 50 μg/mL. Cell viability was determined using the live/dead assay, which employed Calcein-AM and propidium iodide (PI) as fluorescent indicators to distinguish live cells (stained green by Calcein-AM) from dead cells (stained red by PI). The data demonstrated that UVB exposure had led to a notable increase in PI-positive cells, signifying elevated cell mortality. However, treatment with rFLG2, particularly at the 50 μg/mL concentration, significantly reduced UVB-induced cell death (Figure 4a,b).

DCFH-DA is a dye that can be converted into the fluorescent compound DCF by cellular esterases and is used to quantitatively detect intracellular reactive oxygen species (ROS). The escalation in DCF fluorescence following UVB treatment confirmed a surge in the ROS levels compared to the non-exposed controls. In contrast, the introduction of rFLG2 substantially decreased ROS concentrations within the UVB-treated cell population, alleviating oxidative stress (Figure 4c,d). Consistently, considering the established association between ROS and inflammation, the expression of the pro-inflammatory cytokines IL-1β and IL-10 was evaluated. The results indicated that UVB exposure significantly upregulated the expression of these cytokines. However, pretreatment with rFLG2 effectively inhibited this upregulation, suggesting a reduction in the UVB-induced inflammatory response (Figure 4e,f).

Overall, these findings indicate that rFLG2 functioned as a protective agent against UVB-induced cellular damage in HaCaT cells by reducing ROS production and downregulating the expression of critical inflammatory cytokines.

### 3.5. Dermal Injection of rFLG2 Mitigates UVB-Induced Epidermal Hyperplasia in Mice

**Figure 5:**
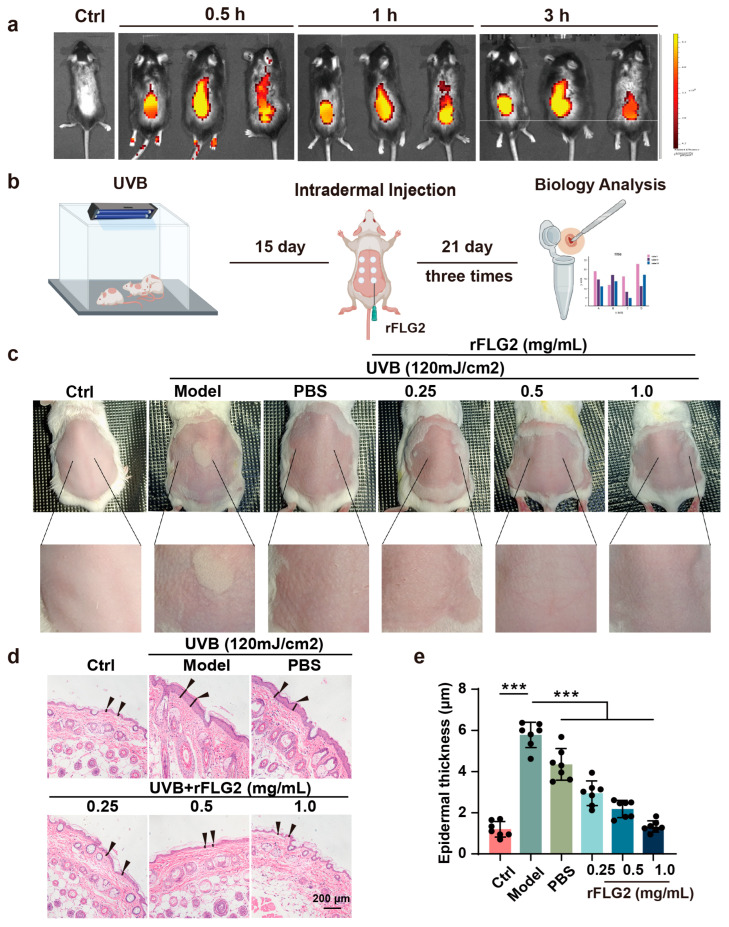
Dermal injection of rFLG2 mitigates UVB-induced epidermal hyperplasia in mice. (**a**) In vivo imaging of Cy5.5-labeled rFLG2 at different times after subcutaneous injection. (**b**) Schematic diagram of the animal experiment. (**c**) Representative images of the morphological changes from each group. (**d**) Representative micrographs of H&E-stained skin tissue sections. Scale bar represents 200 μm. Arrows indicate epidermal thickness. (**e**) Statistics of epidermal thickness in mice (*n* = 7). Values are shown as mean ± SD. *** *p* < 0.001.

To investigate the role of rFLG2 on the epidermal layer in vivo, we first traced its distribution post-administration. To enable fluorescent tracing, rFLG2 was tagged with Cy5.5. Upon dermal injection, rFLG2 localized in the dorsal subcutaneous areas of mice, as revealed by ex vivo fluorescent imaging (Figure 5a).

Subsequently, we established a UVB-induced epidermal barrier damage model by subjecting mice to a dosage of 120 mJ/cm^2^ of UVB radiation for 2 h daily for 15 days, with untreated skin serving as the normal control (Figure 5b). For the therapeutic investigation, mice received injections of various rFLG2 concentrations (0.25 mg/mL, 0.5 mg/mL, 1.0 mg/mL) or PBS as a vehicle control into the dorsal skin for three weeks preceding UVB exposure, with weekly dosing intervals. Both the UVB-irradiated model group and the PBS-treated group displayed visible symptoms, such as erythema, skin roughness, and wrinkle formation. However, these manifestations were notably reduced in the groups treated with rFLG2, at all tested concentrations (Figure 5c). UVB irradiation is widely recognized to induce an increase in epidermal thickness. The untreated control group maintained an epidermal thickness of 1.08 μm, while the model group exhibited a thickness of 5.75 μm, and the PBS-treated group showed a thickness of 4.21 μm. Remarkably, rFLG2 treatment effectively prevented UVB-induced epidermal thickening, with the 1.0 mg/mL rFLG2 group showing a similar epidermal thickness (1.35 μm) to that of the untreated control (Figure 5d,e). These results supported the protective effect of rFLG2 against UVB-induced epidermal hyperplasia.

### 3.6. Dermal Injection of rFLG2 Facilitated Epidermal Differentiation and Accelerated Recovery of UVB-Disrupted Epidermal Barrier

**Figure 6:**
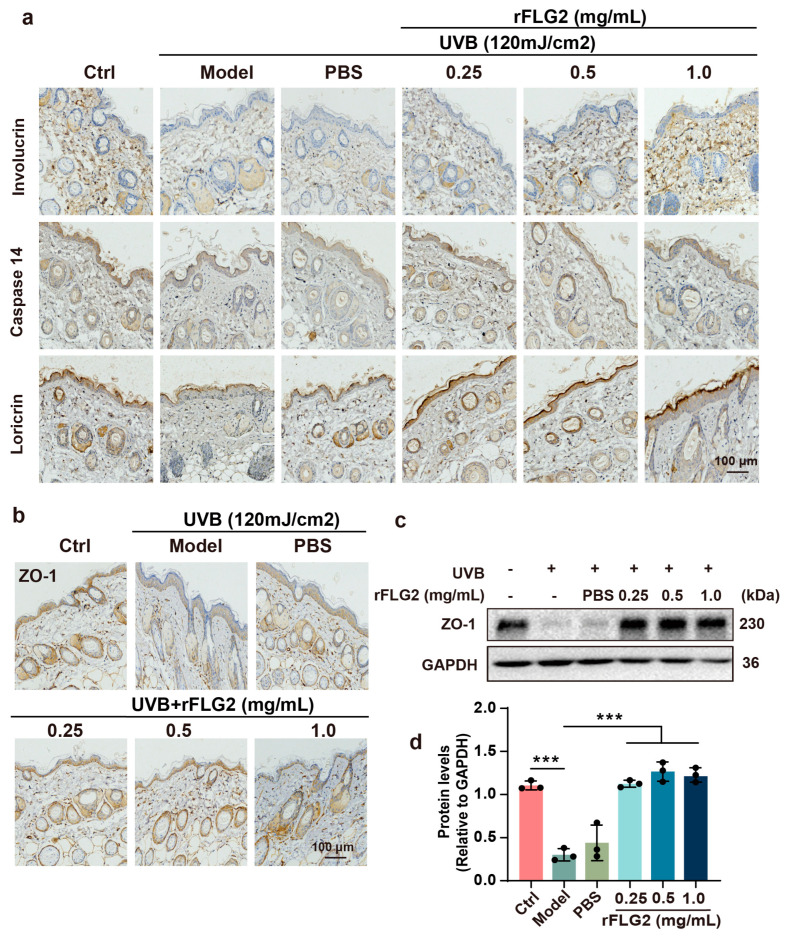
rFLG2 redistributes the expression of barrier proteins in vivo. (**a**,**b**) Immunohistochemical staining of involucrin, loricrin, caspase14, and ZO-1 in skin equivalents from mouse. Images are representative of groups. Scale bar represents 100 μm. (**c**,**d**) Protein level of ZO-1 in dorsal skin of UVB-irradiated mice was analyzed by Western blot (*n* = 3). GAPDH antibody was used as control. Values are shown as means ± SD. *** *p* < 0.001.

Next, we performed immunostaining for epidermal differentiation markers in the dorsal skin of unexposed and UVB-exposed mice. After UVB exposure, there was a noticeable decrease in the staining intensity for differentiation markers, including involucrin, loricrin, and caspase 14, in the granular layer of the epidermis. However, these changes were alleviated with the administration of rFLG2. Concentrations of rFLG2 at 0.5 mg/mL and 1.0 mg/mL upregulated the expression levels of involucrin, loricrin, and caspase 14, comparable to those of the unexposed control group (Figure 6a).

The effect of rFLG2 on the regulation of the TJ-related protein ZO-1 was detected by immunohistochemistry and Western blot. UV irradiation markedly reduced the expression levels of ZO-1, which were subsequently increased by the treatment with rFLG2 (Figure 6b–d). In summary, rFLG2 promoted the expression of proteins associated with epidermal differentiation and barrier function, effectively countering the UVB-induced impairment of the TJ barrier function in keratinocytes.

### 3.7. Dermal Injection of rFLG2 Alleviated UVB-Induced Oxidative Stress and Inflammatory Response in Mice

**Figure 7:**
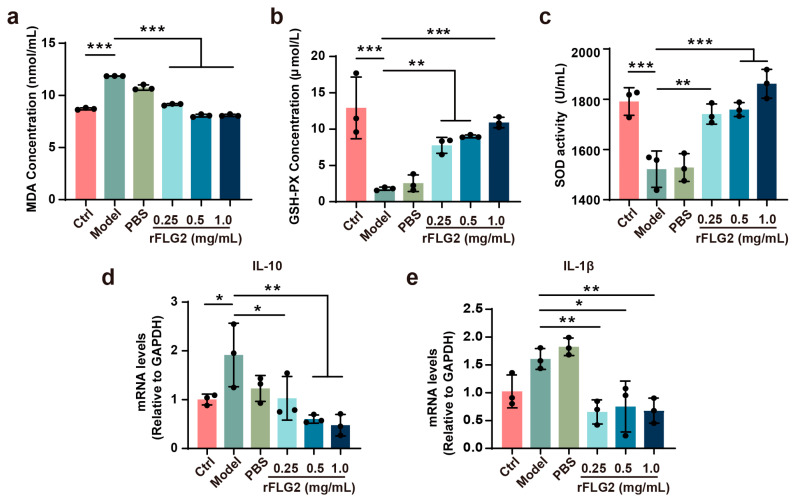
rFLG2 significantly inhibits UVB-induced oxidative stress and inflammatory responses in mice. (**a**) Lipid peroxidation in the dorsal skin in each group after rFLG2 treatment was measured by quantifying malondialdehyde (MDA) (*n* = 3). (**b**) GSH-PX activity in the dorsal skin after rFLG2 treatment (*n* = 3). (**c**) Superoxide dismutase (SOD) activity in the dorsal skin after rFLG2 treatment (*n* = 3). (**d**,**e**) mRNA expressions of *Il-10* and *Il-1β* in skin was measured by RT-qPCR and normalized to GAPDH levels (*n* = 3). Values are shown as means ± SD. * *p* < 0.05, ** *p* < 0.01, *** *p* < 0.001.

UVB exposure typically induces oxidative stress and inflammatory responses in mice, so we measured the activities of SOD, GSH-PX, and MDA in the skin. As expected, a significant UVB exposure increased the level of MDA from 8.8 nM in normal skin to 11.8 nM and 10.9 nM in the model and PBS groups, respectively. However, rFLG2 treatment effectively prevented the rise in MDA levels post-UVB irradiation, with the levels after treatment being similar to those in normal skins (Figure 7a). In parallel, SOD and GSH-PX activities in dorsal skin tissues were markedly reduced after UVB exposure. Treatment with rFLG2 at a concentration of 1 mg/mL essentially restored the activities of SOD (1790 U) and GSH-PX (12.33 μM) to levels observed in the control group (Figure 7b,c).

Additionally, the expression levels of the pro-inflammatory cytokines *Il-10* and *Il-1β* significantly increased following UVB exposure. In contrast, their expression was substantially reduced with rFLG2 treatment (Figure 7d,e). Taken together, rFLG2 effectively attenuated UVB-induced oxidative stress in vivo.

### 3.8. Dermal Injection of rFLG2 Attenuated UVB-Induced Collagen Degradation and Alleviated Photodamage In Vivo

**Figure 8:**
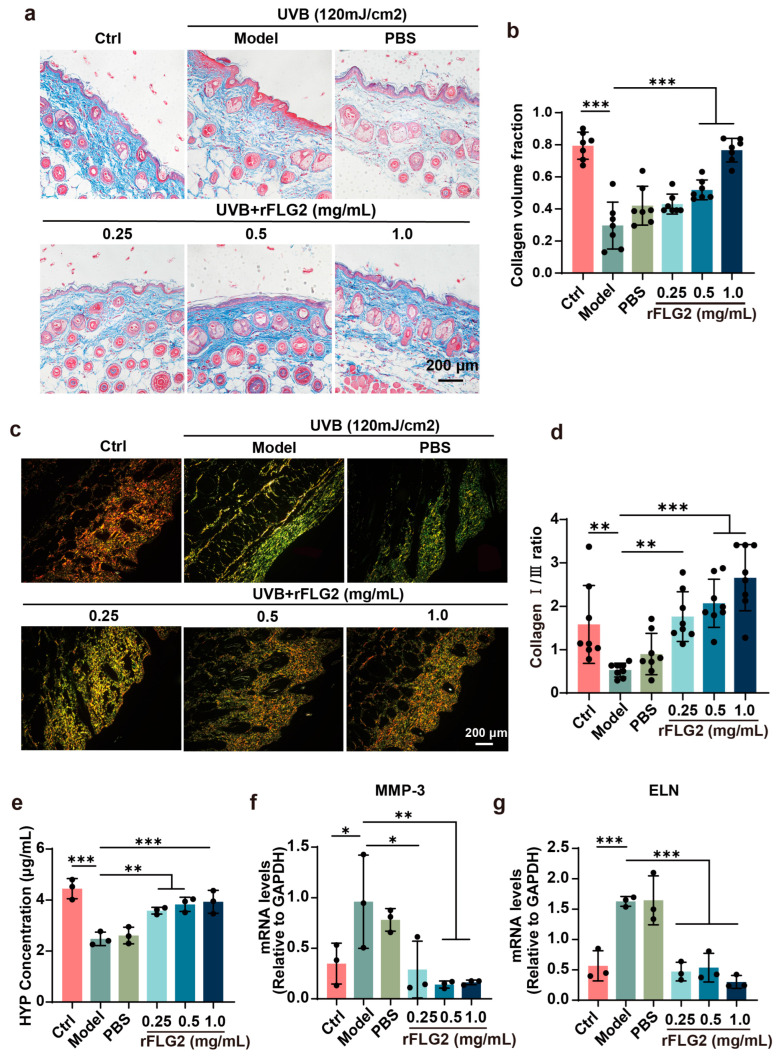
rFLG2 inhibits UVB-induced collagen depletion in mice. (**a**,**b**) Masson staining was used to detect the content of collagen fibers in the skin of each group of mice (*n* = 7). Scale bar represents 200 μm. (**c**,**d**) Picrosirius red staining of the dorsal skin (*n* = 7). Scale bar represents 200 μm. (**e**) HYP activity in the dorsal skin after rFLG2 treatment was quantified (*n* = 3). (**f**,**g**) Relative transcript levels of *Mmp-3* and *elastin*-related collagen genes were determined by RT-qPCR (*n* = 3). mRNA relative levels were normalized to GAPDH levels. Values are shown as means ± SD (*n* = 3). * *p* < 0.05, ** *p* < 0.01, *** *p* < 0.001.

Collagen destruction is thought to underlie the characteristic alterations in the appearance of aged skin [27]. To evaluate the effect of rFLG2 on collagen fibril integrity in murine skin, Masson’s trichrome staining was performed. In the model group, the collagen fibers were observed to be fragmented and disorganized. In contrast, treatment with rFLG2 notably improved the structural integrity of the epidermis, particularly at concentrations of 1.0 mg/mL (Figure 8a,b). The combination of Sirius red staining and polarized light microscopy enabled the visualization and quantification of type I and type III collagens, depicted in red and green, respectively. This methodology revealed a sparse distribution of type I and III collagen in the model group when compared to the control, indicating that UV radiation had detrimental effects on the dermis. Treatment with rFLG2 at 0.5 mg/mL and 1.0 mg/mL concentrations restored the ratio of type I and III collagen to levels comparable to the normal group (Figure 8c,d). Furthermore, the collagen content within the skin was quantified using a hydroxyproline (HYP) assay. The results demonstrated a substantial decrease in collagen levels due to UVB exposure, with HYP levels falling from 4.45 μg/mL to 2.45 μg/mL. Conversely, rFLG2 treatment elevated the HYP levels from 2.45 μg/mL to 3.93 μg/mL, signifying an increase in collagen deposition at concentrations of 0.5 mg/mL and 1.0 mg/mL (Figure 8e).

Photoaging of the skin is predominantly characterized by collagen degradation and abnormal elastin remodeling. An increase in MMPs (matrix metalloproteinases) and elastin levels is one of the primary drivers of these changes. As expected, the levels of *elastin* in both the UVB-irradiated and PBS-treated groups were triple those of the control group, suggesting that UVB-induced abnormal elastin accumulation. Moreover, the *Mmp-3* levels in these groups rose by 3.67-fold and 2.16-fold, respectively. Remarkably, rFLG2 treatment at concentrations of 0.5 mg/mL and 1.0 mg/mL significantly reduced the expression of *elastin* and *Mmp-3* genes compared to both the model and PBS-treated groups (Figure 8f,g). These findings suggested that rFLG2 attenuated UVB-induced collagen degradation and alleviated photoaging in the skin.

## 4. Discussion

In this study, we observed that UVB radiation leads to a decrease in the expression of FLG2 in the epidermis, which consequently precipitates skin barrier impairment. Furthermore, we examined the efficacy of rFLG2 in improving the repair function of the epidermal barrier. rFLG2 increased the expression of differentiation markers in the epidermal barrier to facilitate epidermal differentiation and mitigate UV-induced epidermal hyperplasia in mice. Furthermore, rFLG2 accelerated the recovery of the epidermal barrier by preventing collagen degradation, regulating oxidative stress, and diminishing inflammatory cyto-kine production. These findings confirmed the important role of FLG2 in maintaining skin health amidst environmental challenges.

Chronic exposure to the UV radiation from sunlight can affect epidermal differentiation and epidermal barrier function, contributing to skin photoaging [28]. The external application of UVB irradiation to the skin can lead to epidermal changes such as dryness, desquamation, and hyperkeratosis [29,30]. The stability of the epidermal barrier relies on a balance between skin cell proliferation and differentiation. Repairing the skin barrier is one of the effective strategies for treating photoaging [31,32]. It has been noted that 1-(2-Hydroxyethyl)-2-imidazolidinone (a heparanase and matrix metalloproteinase inhibitor) [33], polysaccharides [34], and Leucine-rich repeat LGI family member 3 (LGI3) can promote keratinocyte differentiation [35], improve epidermal basement membrane structure, and enhance epidermal barrier function. In our study, we found that UVB exposure significantly reduced the mRNA expression of the epidermal differentiation markers involucrin and loricrin compared to the control group. Conversely, treatment with rFLG2 notably increased the expression of these genes. In addition, the expression of caspase14, an enzyme activated in fully differentiated keratinocytes [36], was upregulated following rFLG2 treatment, suggesting that rFLG2 fosters keratinocyte differentiation. Interestingly, while the treatment with rFLG was capable of increasing the expression of the FLG gene, the impact was not statistically significant. This suggests that the function of rFLG2 to improve epidermal barrier dysfunction is attributed to its direct action and is independent of FLG expression.

Furthermore, we found that rFLG2 boosts the expression of occludin and ZO-1, thereby enhancing the TJ barrier function and supporting keratinocyte recovery after UV-induced TJ damage. ZO-1 is an essential component of TJs, found in the basal and suprabasal layers of the epidermis [37], while the TJ barrier in the stratum granulosum is vital for complete stratum corneum formation and barrier functionality. In patients with atopic dermatitis, a decrease in claudin-1 expression leads to TJ and epidermal barrier dysfunction, which results in inflammatory changes within the human epidermis [38]. Increased expressions of claudin-1 and ZO-1 have been found to elevate the TEER and enhance the TJ barrier function [39,40]. Our results suggested that rFLG2 increases ZO-1 and occludin expression, which in turn raises TEER and enhances the TJ barrier function. Consequently, rFLG2 promoted keratinocyte differentiation and enhanced TJ barrier function, which supported the repair of UV-induced skin barrier defects. On the other hand, while TEWL and SCH are important parameters, our study primarily focused on the expression levels of barrier-related proteins to assess the accelerated recovery of the UVB-disrupted epidermal barrier by rFLG2. The presence of hair in the murine model could potentially interfere with the accurate measurement of TEWL and SCH, as hair can alter the rate of skin surface water evaporation and the hydration of the stratum corneum [41]. This may result in measurements that do not directly correspond to human skin conditions. Therefore, our assessment of rFLG2’s effect has centered on measuring barrier-related protein expression levels. While this approach provides valuable insights, we acknowledge its limitations, and in future studies, we will consider using hairless models or ex vivo human skin to better simulate human conditions and further validate the therapeutic potential of rFLG2.

UVB radiation has been widely reported to induce an inflammatory response, leading to oxidative stress and collagen degradation, consequently compromising the integrity of the skin barrier [42,43]. Prior research has illustrated that natural components may exhibit photoprotective properties, such as enhanced skin elasticity, improved hydration and texture, and reduction in wrinkles [44,45]. These positive outcomes are largely attributed to their antioxidant capacities and their roles in modulating UV-induced skin inflammation and aging processes. According to our research, rFLG2 significantly reduces the generation of reactive oxygen species (ROS) and the expression of pro-inflammatory cytokines, such as IL-10 and IL-1β, which helps to mitigate the oxidative damage caused by UVB exposure. Moreover, the elevated levels of ROS associated with UV radiation are known to induce lipid peroxidation, as indicated by increased levels of MDA, and to impair the activity of antioxidant enzymes such as GSH-PX and SOD. Our findings demonstrated that post-UVB irradiation, there is a significant rise in MDA levels accompanied by a notable reduction in both GSH-PX and SOD. However, following rFLG2 treatment, we observed a substantial decrease in MDA levels and a significant increase in GSH-PX and SOD, with values approaching near-normal levels.

Additionally, MMPs play a pivotal role in the UV-induced degradation of proteins, with MMP-3 being instrumental in breaking down type III and IV collagen and gelatin, as well as in activating MMP-1 and other MMP family members [46,47]. The degradation of collagen disrupts the natural dynamic equilibrium of the extracellular matrix (ECM), leading to the structural remodeling and functional impairment of the skin. Our analyses included measuring the collagen content, and the results indicated a significant increase in the collagen levels following rFLG2 intervention. The observed decrease in inflammatory cytokines and the prevention of collagen degradation in response to rFLG2 treatment suggest that the targeted regulation of FLG2 genes could possess the capability to counteract photoaging. This evidence provides compelling support for the beneficial role of rFLG2 in the maintenance of skin health against the deleterious effects of UVB radiation.

Both UVB and UVA play significant roles in photoaging. As the energy produced by UV decreases with increasing wavelength, UVB poses a greater threat to the epidermis than UVA [48,49]. Nevertheless, UVB mainly damages the epidermis and superficial dermis, whereas UVA possesses greater penetrating capabilities, allowing it to penetrate the skin deeply and substantially contribute to the deterioration of the dermal structure and photoaging [50,51]. However, UVA and UVB have some overlapping dermatological effects. Both UVA and UVB have been shown to be responsible for erythema, oxidative stress, photo-immunosuppression, DNA damage, and skin cancer [52,53]. Mechanistically, UVA also increases the levels of MMP1 and MMP3 and promotes the degradation of type I and III collagen and elastin [54,55]. Our study has demonstrated the effectiveness of rFLG in ameliorating UVB-induced oxidative stress, collagen degradation, reduced elastin expression, and upregulation of MMP-3. Based on this, it is possible for rFLG to improve UVA-induced photoaging and skin damage, which deserves further exploration. Additionally, while these findings are promising, they stem from a murine model that, although valuable, does not fully replicate human skin physiology. Future studies should extend to long-term clinical trials to fully evaluate the safety and efficacy of rFLG2 in human populations. Enhancing our understanding of optimized delivery methods and rFLG2’s impact on various skin types and conditions will also be crucial.

**Figure 9:**
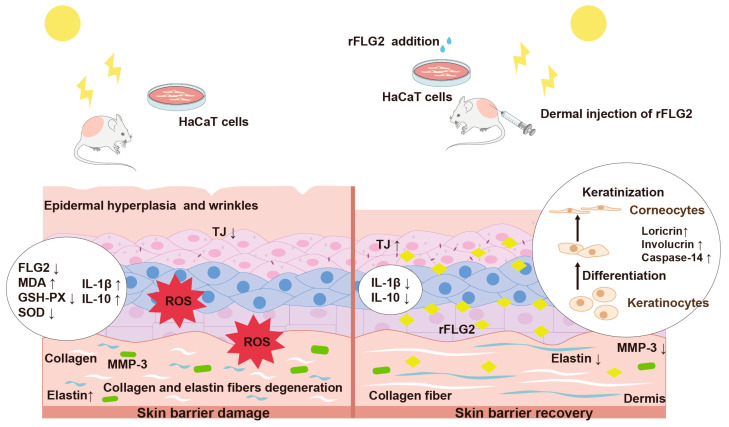
Mechanisms involved in protective effects of rFLG2. The up arrows represent increased protein expression, while the down arrows represent decreased protein expression.

In summation, rFLG2 presents itself as a potent agent for the prevention and amelioration of skin damage inflicted by UVB radiation within the field of dermatological care. By bolstering cellular resilience and mitigating inflammatory responses, rFLG2 aids in epidermal recuperation and may serve to decelerate the senescent processes precipitated by photoaging (Figure 9). These results imply that targeting FLG2 could be instrumental in forestalling the initiation and progression of a multitude of skin disorders, including atopic dermatitis and intrinsic skin aging, by restoring the compromised barrier function of the skin.

## 5. Conclusions

Our study revealed that UVB radiation has a detrimental effect on HaCaT keratinocytes by triggering senescence, suppressing FLG2 expression, and compromising barrier integrity. The application of rFLG2 has been found to improve epidermal barrier function restoration, reduce UVB-induced oxidative damage, and lower pro-inflammatory cytokine expression both in vitro and in vivo. Additionally, rFLG2 has proven effective in inhibiting collagen degradation, which is often accelerated by excessive UV exposure. These findings suggested that rFLG2 may emerge as a pivotal element in skincare routines aimed at safeguarding and repairing the epidermis from UVB-induced harm.

## Figures and Tables

**Table 1:** Genes and primers used for the qRT-PCR analyses.

Gene Name	Sequence	Gene Accession Number
h-IL-1β-F	CAGGCTGCTCTGGGATTCTC	NM_000576.3
h-IL-1β-R	CCTGGAAGGAGCACTTCATCT	NM_000576.3
h-IL-10-F	GCCTAACATGCTTCGAGATC	NM_000572.3
h-IL-10-R	TGATGTCTGGGTCTTGGTTC	NM_000572.3
h-Involucrin-F	TCCTCCAGTCAATACCCATCAG	NM_005547.4
h-Involucrin-R	CAGCAGTCATGTGCTTTTCCT	NM_005547.4
h-Caspase14-F	TTCCGAAGAAGACCTGGATG	NM_012114.3
h-Caspase14-R	TGGGGTCTCTTTTCATGGTG	NM_012114.3
h-Loricrin-F	TCATGATGCTACCCGAGGTTTG	NM_000427.3
h-Loricrin-R	CAGAACTAGATGCAGCCGGAGA	NM_000427.3
h-FLG2-F	CACTGAGCAAGGGTGAACTAAA	NM_001014342.3
h-FLG2-R	ACCACGCCTATGCTTCTTTGA	NM_001014342.3
h-GAPDH-F	CACCATCTTCCAGGAGCGAG	NM_001256799.3
h-GAPDH-R	AGAGGGGGCAGAGATGATGA	NM_001256799.3
m-*Il-1β*-F	GCCACCTTTTGACAGTGATGAG	NM_008361.4
m-*Il-1β*-R	GACAGCCCAGGTCAAAGGTT	NM_008361.4
m-*Il-10*-F	GGAGGGGTTCTTCCTTGGGA	NM_010548.2
m-*Il-10*-R	TGAGCTGCTGCAGGAATGAT	NM_010548.2
m-*Elastin*-F	GCTGGAGGTTTAGTGCCTGG	NM_007925.4
m-*Elastin*-R	GCTCCGTATTTGGCAGCTTT	NM_007925.4
m-*Mmp-3*-F	ACATGGAGACTTTGTCCCTTTTG	NM_010809.3
m-*Mmp-3*-R	TTGGCTGAGTGGTAGAGTCCC	NM_010809.3
m-*Gspdh*-F	TGTGTCCGTCGTGGATCTGA	NM_001289726.2
m-*Gapdh*-R	CCTGCTTCACCACCTTCTTGA	NM_001289726.2

## Data Availability

The data presented in this study are available in the article.
